# ABA-Mediated ROS in Mitochondria Regulate Root Meristem Activity by Controlling *PLETHORA* Expression in *Arabidopsis*

**DOI:** 10.1371/journal.pgen.1004791

**Published:** 2014-12-18

**Authors:** Li Yang, Jing Zhang, Junna He, Yingying Qin, Deping Hua, Ying Duan, Zhizhong Chen, Zhizhong Gong

**Affiliations:** 1 State Key Laboratory of Plant Physiology and Biochemistry, College of Biological Sciences, China Agricultural University, Beijing, China; 2 National Center for Plant Gene Research, Beijing, China; National University of Singapore and Temasek Life Sciences Laboratory, Singapore

## Abstract

Although research has determined that reactive oxygen species (ROS) function as signaling molecules in plant development, the molecular mechanism by which ROS regulate plant growth is not well known. An *ab*a *o*verly sensitive mutant, *abo8-1*, which is defective in a pentatricopeptide repeat (PPR) protein responsible for the splicing of *NAD4* intron 3 in mitochondrial complex I, accumulates more ROS in root tips than the wild type, and the ROS accumulation is further enhanced by ABA treatment. The *ABO8* mutation reduces root meristem activity, which can be enhanced by ABA treatment and reversibly recovered by addition of certain concentrations of the reducing agent GSH. As indicated by low *ProDR5:GUS* expression, auxin accumulation/signaling was reduced in *abo8-1*. We also found that ABA inhibits the expression of *PLETHORA1* (*PLT1*) and *PLT2*, and that root growth is more sensitive to ABA in the *plt1* and *plt2* mutants than in the wild type. The expression of *PLT1* and *PLT2* is significantly reduced in the *abo8-1* mutant. Overexpression of PLT2 in an inducible system can largely rescue root apical meristem (RAM)-defective phenotype of *abo8-1* with and without ABA treatment. These results suggest that ABA-promoted ROS in the mitochondria of root tips are important retrograde signals that regulate root meristem activity by controlling auxin accumulation/signaling and *PLT* expression in *Arabidopsis*.

## Author summary

Abscisic acid (ABA) plays crucial roles in plant growth and development, and also in plant responses to abiotic and biotic stresses. ABA can stimulate the production of reactive oxygen species (ROS) that act as signals in low concentrations, but as cell-damaging agents in high concentrations. A mutation in *ABO8*, encoding a pentatricopeptide repeat (PPR) protein responsible for the splicing of *NAD4* intron 3, leads to hypersensitivity to ABA in root growth, and root tips of the *abo8-1* mutants accumulate more ROS than those of the wild type; this accumulation of ROS in *abo8-1* root tips is enhanced by ABA treatment. We also found that auxin signaling and/or accumulation is greatly reduced in root tips of the *abo8-1* mutants. Addition of the reducing agent GSH to the growth medium partially recovers the root hypersensitivity to ABA, and also the ABA-inhibited expression of *PLT1/2* in *abo8-1*. Furthermore, the inducible expression of *PLT2* largely rescues the root growth defect of *abo8-1* with and without ABA treatment. Our results reveal the important roles of ROS in regulating root meristem activity in the ABA signaling pathway.

## Introduction

Plant growth and development are greatly influenced by the accumulation of reactive oxygen species (ROS), which are produced under environmental stresses such as water deficiency and high salinity [1], [2], [3], [4]. Although hazardous to cells at high concentrations, ROS at low concentrations are important signaling molecules that regulate stomatal movement, prevent pathogen invasion, promote programmed cell death, and redirect plant growth [5], [6], [7], [8]. In addition to linking with stress hormones such as ethylene, salicylic acid, jasmonic acid, and abscisic acid (ABA) [9], ROS also have cross-talk with growth hormones such as gibberellin [10], [11], cytokinins and auxin [1], [4].

The connection between ROS and auxin has been explored in the regulation of development of the root apical meristem. Ascorbate (ASC) and glutathione (GSH) are the two main antioxidants in plants. Dehydroascorbate treatment, which reduces ASC, abolished auxin maximum [1]. Root growth is promoted by the addition of micromolar concentrations of GSH [12], while depletion of GSH through BSO treatment [12] or in the glutamylcysteine synthetase (the first enzyme for glutathione biosynthesis) mutant (cadmium sensitive2 (*cad2)/root meristemless1(rml1)*) [13], [14] or in *miao* mutant (a weak mutation occurring in plastid-localized glutathione reductase2 (GR2)) [15] retards root growth with perturbation of auxin signaling and response in *Arabidopsis*. The triple mutant with disruption of two NADP-linked thioredoxin reductase gene (NTRA and NTRB) and CAD2/RML1 results in altered auxin homeostasis and reduced root growth [16]. The auxin-receptor mutants *tir1 afb2* and *tir1 afb3* are more resistant to oxidative stress and produce less ROS under salt treatment than the wild type [17]. Researchers recently found that ABA promotes ROS production through both plasma membrane-associated NADPH oxdixases [18] and mitochondria [19]. The accumulation of ROS in the mutant of *ABO6* (*ABA O*verly sensitive 6 encodes a DEXH box RNA helicase that regulates the splicing of genes in complex I) disrupts auxin homeostasis and inhibits root growth under ABA treatment [19]. The oxidation of active indole-3-acetic acid (IAA) into the low activity 2-oxindole-3-acetic acid (OxIAA) mainly occurs in the root apex in *Arabidopsis* and should play crucial roles in auxin homeostasis for regulating root growth [20].

Previously, we cloned *ABO5* (*AT1G51965*), which encodes a pentatricopeptide repeat protein required for *cis*-splicing of mitochondrial *NAD2* intron 3 [21], and *ABO6* [19]. In this study, we cloned *ABO8*, which encodes another pentatricopeptide repeat protein that is involved in regulating the splicing of mitochondrial complex I *NAD4* intron 3. *ABO8* is highly expressed in root tips and lateral root primordia. The *abo8-1* mutant accumulates more ROS than the wild type in its root tips. The expression of *PLETHORA1* (*PLT1*) and *PLT2* is reduced in *abo8-1*, and inducible expression of *PLT2* can largely rescue the reduced root meristem activity in *abo8-1* with and without ABA treatment. These results suggest that ROS produced in mitochondria are important retrograde signals for controlling root meristem activity under environmental stress.

## Results

### The *abo8* mutants show retarded growth and are more sensitive to ABA than the wild type

**Figure 1:**
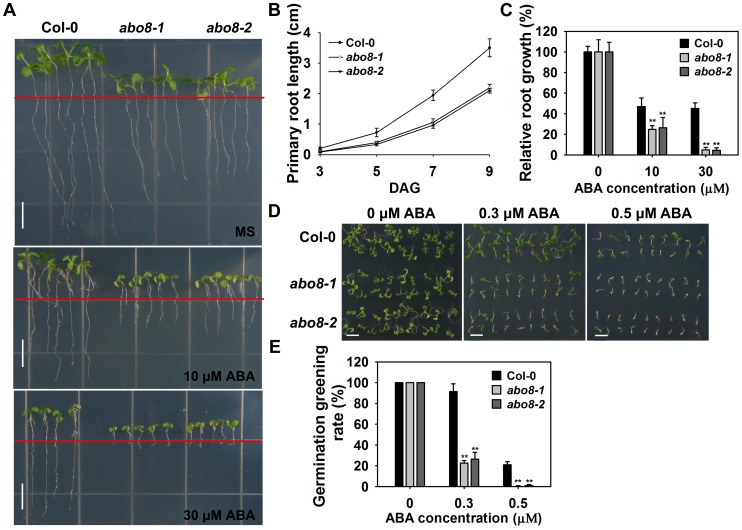
*abo8* mutants are hypersensitive to ABA in seed germination and root growth. A. The root growth phenotype of *abo8* mutants on MS medium or MS medium supplemented with different concentrations of ABA. Four-day-old seedlings grown on MS were transferred to MS medium containing 0, 10, or 30 µM ABA for 5 days before they were photographed. Bars = 5 mm. B. Statistical analysis of the root length in different growing times. C. Relative root growth. Root length is expressed relative to that of the wild type or *abo8* mutants without ABA. In (B) and (C), three independent experiments were done with similar results, each with three biological repeats. Five roots from one plate were measured for each repeat. Values are means ±SE, n = 3. **: P<0.01. D. Seed germination greening of *abo8* mutants after 7 days on MS medium supplemented with 0.0, 0.3, or 0.5 µM ABA. Bars = 2 mm. E. Statistical analysis of seed germination greening rate in (D). Three independent experiments were done, each with three replicates (from three different plates). About 24 seeds were counted for each plate. Values are means ±SE, n = 3. **: P<0.01.

During a genetic screening for ABA overly sensitive mutants in root growth [19], [21], we isolated a novel mutant, *aba-overly sensitive 8-1* (*abo8-1*). The F1 seedlings of *abo8-1* crossed with the wild type showed the wild type phenotypes, indicating that *abo8-1* is a recessive mutation. *abo8-1* was backcrossed with the wild type for 4 times before performing further physiological analyses. A T-DNA insertion line, *abo8-2*, was obtained from the Arabidopsis Stock Center after we cloned *ABO8* gene (see later for detail information). *abo8-1* and *abo8-2* had similar growth phenotypes. Relative to the wild type, *abo8-1* and *abo8-2* mutants had shorter roots and were smaller under normal growth conditions (Fig. 1A, 1B). ABA-inhibition of root growth and germination was greater in *abo8-1* and *abo8-2* than in the wild type (Fig. 1A–E). These results demonstrate that mutations in *ABO8* retard plant growth and causes ABA hypersensitivity in *Arabidopsis*.

### *ABO8* encodes a pentatricopeptide repeat protein that is highly expressed in root tips and lateral root primordia

**Figure 2:**
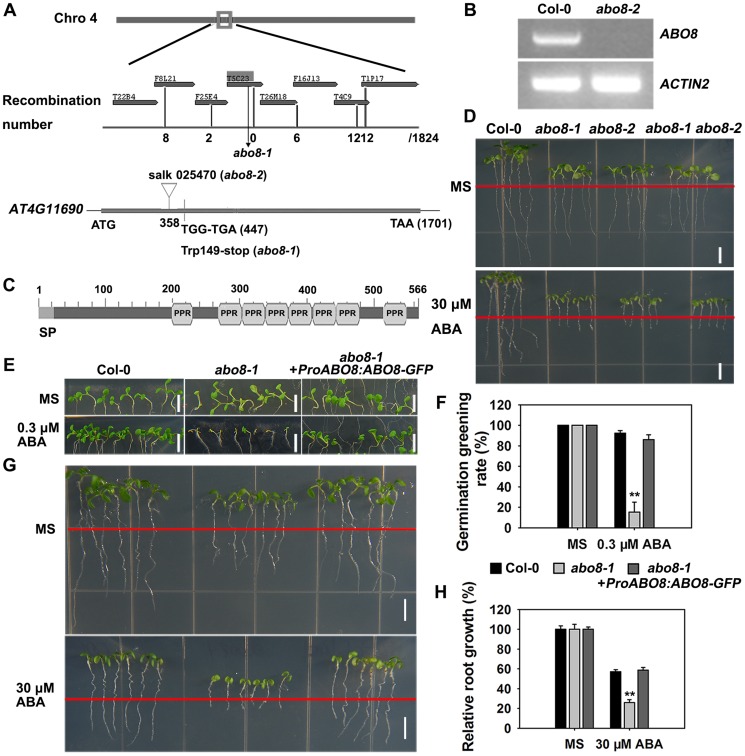
Map-based cloning of *ABO8*. A. *ABO8* was mapped to chromosome 4. A point mutation from G447 to A447 (counting from the first putative ATG in *AT4G11690*) was identified in *abo8-1*. A T-DNA insertion line SALK_025470 (*abo8-2*, T-DNA is inserted in position +358) was obtained from the Arabidopsis Biological Resource Center. B. RT-PCR analysis of *ABO8* transcripts in *abo8-2* using primers flanking the T-DNA insertion. Expression of the *ACTIN2* gene was used as control. C. The protein structure of ABO8. SP: signal peptide; PPR: pentatricopeptide repeat. D. Genetic analysis of *abo8-1* and *abo8-2* for sensitivity of root growth to ABA. Four-day-old seedlings grown on MS medium were transferred to MS medium containing 0 or 30 µM ABA. *abo8-1 abo8-2*: F1 seedlings of *abo8-1* crossed with *abo8-2*. Bars = 5 mm. E. Seed germination greening analysis of *abo8-1* complemented with an *ABO8* promoter driving *ABO8* cDNA fused with *GFP* cDNA (*ProABO8:ABO8-GFP*) on MS medium containing 0.0 or 0.3 µM ABA. Bars = 5 mm. F. Statistical analysis of seed germination greening rate of the complemented *abo8-1* in (E). Three independent experiments were done, each with three replicates (from three different plates). About 24 seeds were counted for each plate. Values are means ±SE, n = 3. **: P<0.01. G. Roots of *abo8-1* complemented with *ProABO8:ABO8-GFP*. Four-day-old seedlings were transferred to MS medium containing 0 or 30 µM ABA. Bars = 5 mm. H. Statistical analysis of root length in (G). Root length is expressed relative to that of the wild type or *abo8-1* without ABA. Three independent experiments were done with similar results, each with three biological repeats. Five roots from one plate were measured for each repeat. Values are means ±SE, n = 3. **: P<0.01.

We used map-based cloning to identify *ABO8*. Those seedlings from the F2 progeny of *abo8-1* crossed with L*er* showing hypersensitivity to ABA in root growth were selected and used for mapping. The *abo8-1* mutation was narrowed to chromosome 4 on BAC clone T5C23 (Fig. 2A). The sequencing of candidate genes revealed a point mutation in *AT4G11690*, which changes G447 to A447 (counting from the first putative ATG of *AT4G11690*) and causes an amino acid change from Trp to a stop code (Fig. 2A). The produced putative truncated protein in *abo8-1* should have no function. In *abo8-2*, a T-DNA was inserted at the position 358 (from the first putative ATG) (Fig. 2A). No transcript was detected in *abo8-2* using primers flanking the T-DNA insertion (Fig. 2B). *ABO8* encodes a P-type pentatricopeptide repeat (PPR) domain protein, which contains a classic tandem array of 35 amino acid PPR motifs, typically with a proline at the end of each motif (Fig. 2C) [22]. The F1 seedlings of *abo8-1* crossed with *abo8-2* showed a similar ABA hypersensitivity as *abo8-1* or *abo8-2* in root growth (Fig. 2D). *ABO8* promoter driving *ABO8* cDNA fused with *GFP* in frame (*ProABO8:ABO8-GFP*) complemented the ABA-hypersensitive seed germination and root growth phenotype of *abo8-1* (Fig. 2E–H). These results confirmed that the mutations in *AT4G11690* led to the phenotypes of the *abo8* mutants.

**Figure 3:**
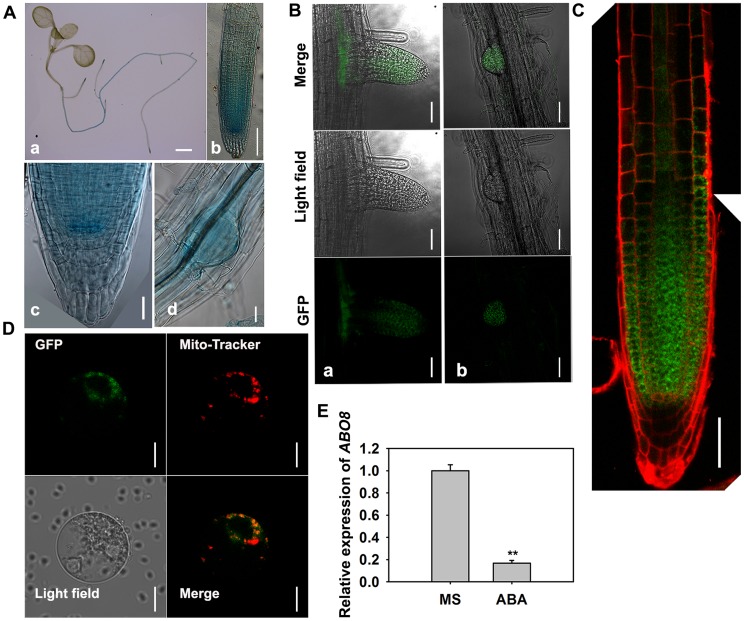
The expression and localization of ABO8. A. The expression pattern of *ProABO8:GUS* in the whole seedling and roots. a, a seedling, bar = 1 mm; b, a primary root, bar = 50 µm; c, an amplified primary root tip, bar = 20 µm; d, a lateral root primordium, bars = 50 µm. B. The expression of *ProABO8:ABO8-GFP* in a lateral root (a, left) and a lateral root primordium (b, right). Bars = 50 µm. C. The expression of *ProABO8:ABO8-GFP* in a primary root. Bars = 50 µm. D. Co-subcellular localization of ABO8-GFP with Mito-Tracker. Bars = 10 µm. E. Expression of *ABO8* is reduced by ABA treatment. Seedlings were treated with or without 30 µM ABA for 8 h, and total RNAs were used for qRT-PCR. *ACTIN2* was used as a control. Three independent experiments were done with similar results, each with three biological replicates. Results shown are from one experiment. Values are means ±SE, n = 3. **: P<0.01.

P-type PPR proteins usually participate in RNA stabilization, processing, splicing, and translation. To determine the expression pattern of *ABO8*, we made transgenic plants carrying *ABO8* promoter:*GUS* (*ProABO8:GUS*). GUS staining indicated that *ABO8* was highly expressed in root tips and lateral root primordia (Fig. 3A). Expression patterns were similar for *ProABO8:ABO8-GFP* and *ProABO8:GUS* (Fig. 3B, 3C). We isolated the protoplasts from *ProABO8:ABO8-GFP* transgenic plants and treated the protoplasts with Mito-tracker, which specifically stains mitochondria. Most GFP fluorescence was co-localized with Mito-tracker (Fig. 3D), indicating that ABO8-GFP is localized in the mitochondria. Real-time PCR analysis indicated that the expression of *ABO8* was significantly suppressed by ABA treatment (Fig. 3E).

### ABO8 is required for splicing of the complex I gene *NAD4*

**Figure 4:**
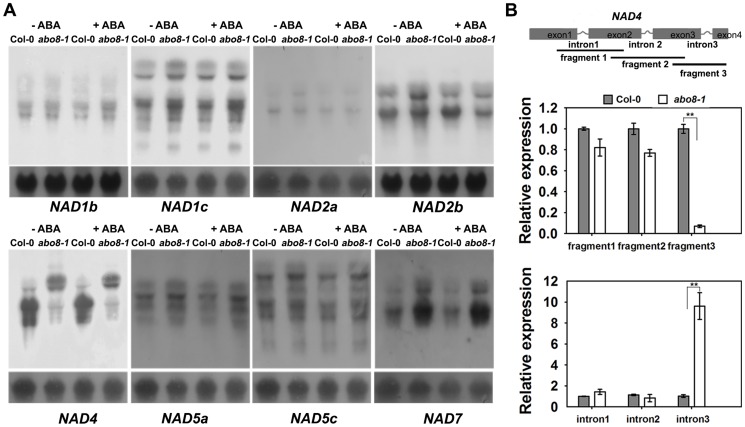
ABO8 regulates the splicing of *NAD4* intron 3. A. Northern blot analyses of the expression of different subunits in complex I. Only *NAD4* showed the splicing defects in *abo8-1*. B. qRT-PCR analysis of different fragments of the *NAD4* transcript. A pair of primers covering different fragments or different introns was used for qRT-PCR. Total RNAs from 10-day-old seedlings were used for qRT-PCR. Three independent experiments were done with similar results, each with three biological repeats. Values are means ±SE from one experiment. **: P<0.01.

In our previous study, we found that the PPR protein ABO5 is responsible for *cis*-splicing of mitochondrial *NAD2* intron 3 [21]. We used northern blot to compare the transcripts of different genes in complex I between the wild type and *abo8-1*. The results indicated that the transcript sizes of all *NAD* genes except *NAD4* are similar for the wild type and *abo8-1* (Fig. 4A) with and without ABA treatment. The *NAD4* transcripts were larger in *abo8-1* than in the wild type, indicating a defect in pre-mRNA splicing in *abo8-1* (Fig. 4A). We designed the primers inside exons that cover the three introns of *NAD4* to determine which intron is affected by the *abo8-1* mutation. Real-time RT-PCR indicated that fragment 3 (covering exons 3 and 4) is greatly reduced in *abo8-1* but that intron 3 (primers inside introns) is significantly increased in *abo8-1* relative to the wild type (Fig. 4B). These results indicate that the *abo8-1* mutation impairs the splicing of the third intron of *NAD4* in complex I.

### *abo8-1* reduces the activity of the electron transport chain in complex I, and accumulates more ROS than the wild type in its root tips

**Figure 5:**
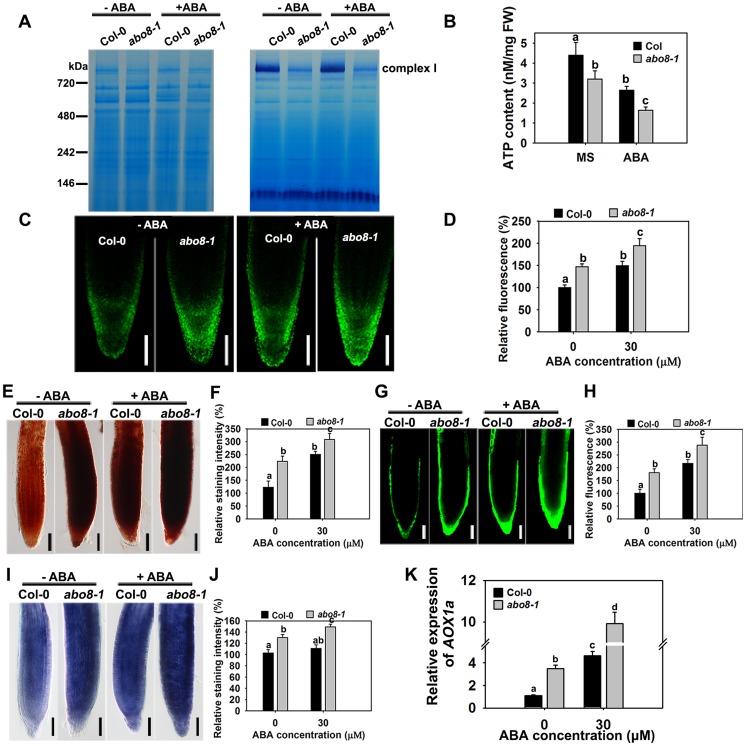
*abo8-1* accumulates more ROS than the wild type in root tips. A. The activity of complex I in the wild type (WT) and *abo8-1*. Crude mitochondria membrane protein extracted from 5-day-old seedlings treated with or without 50 µM ABA for 24 h were separated by Blue Native-PAGE. Coomassie staining (left) indicates protein complexes after electrophoresis; Complex I activity was preformed by in-gel staining with NADH and NBT (nitroblue tetrazolium) for the NADH dehydrogenase activity of Complex I. B. ATP content in WT and *abo8-1* plants. 5-day-old WT and *abo8-1* seedlings treated with or without 50 µM ABA for 24 h were harvested for measurement of ATP content. FW, fresh weight. Means with different letters are significantly different at P<0.01. C. Fluorescence analysis of mitochondrial cpYFP in primary root tips of the wild type and *abo8-1* with or without ABA treatment. Bars = 50 µm. D. cpYFP intensity as determined with AxioVision Rel. 4.8 software. E. DAB staining for H_2_O_2_ in primary root tips of the wild type and *abo8-1* with or without ABA treatment. Bars = 50 µm. F. DAB staining intensity as determined with Adobe Photoshop 3.0 software. G. DCFH-DA staining for H_2_O_2_ in primary root tips of the wild type and *abo8-1* with or without ABA treatment. Bars = 50 µm. H. DCFH-DA staining intensity as determined with AxioVision Rel. 4.8 software. I. NBT staining for superoxide in primary root tips of the wild type and *abo8-1* with or without ABA treatment. Bars = 50 µm. J. NBT staining intensity as determined with Adobe Photoshop 3.0 software. Three independent experiments were done with similar results, each with three replicates, and each replicate with 15–20 roots. Values are means ±SE from one experiment. Means with different letters are significantly different at P<0.01. K. Relative expression levels of *AOX1a* in seedlings treated with or without 50 µM ABA for 5 h in liquid MS. Three independent experiments were done with similar results, each with three biological replicates. Results shown are from one experiment. Values are means ±SE, n = 3. Means with different letters are significantly different at P<0.01.

Impairment of the electron transport chain in complex I usually reduces the NADH dehydrogenase activity, which affects the production of ATP, and increases ROS accumulation in cells. We compared the NADH dehydrogenase activity of complex I between wild type and *abo8-1* by Blue Native-PAGE [23]. Comparing with the wild type, complex I activity was largely reduced in *abo8-1* (Fig. 5A). Consistently, *abo8-1* produced much less ATP than wild type, and ABA treatment further reduced the ATP production in both the wild type and *abo8-1* (Fig. 5B). We introduced a mitochondrial superoxide marker Mito-cpYFP into *abo8-1* to monitor ROS accumulation in roots. The previous results indicated that the strong fluorescence emitted by Mito-cpYFP in mitochondria was correlated with the high oxidation status [19]. Mito-cpYFP fluorescence was highest near root tips and progressively decreased from the outside of the columella cell layer to the inside below the QC (quiescent centre) in both the wild type and *abo8-1*. *abo8-1* emitted stronger fluorescence than the wild type (Fig. 5C, 5D). In comparing the production of H_2_O_2_ (using DAB and DCFH-DA staining) and of superoxide (using NBT staining) in root tips of the wild type and *abo8-1*, we found that the root tips of *abo8-1* accumulated more H_2_O_2_ and superoxide than those of the wild type (Fig. 5E–J). ABA treatment significantly enhanced the production of H_2_O_2_ and superoxide in both *abo8-1* and wild type (Fig. 5E–J). These results suggest that dysfunction of *ABO8* increases ROS production in root tips and that ROS production is enhanced by ABA treatment.

Besides the classical electron transport chain, plants also have alternative NADH dehydrogenases that can maintain the oxidation of matrix NADH in mitochondria when complex I is not working. *ALTERNATIVE OXIDASE1a* (*AOX1a*) is a marker gene in alternative respiratory pathway for mitochondria stress. The expression of *AOX1a* was higher in *abo8-1* than the wild type, and ABA treatment further increased its expression in both *abo8-1* and the wild type, and its expression was still higher in *abo8-1* than in the wild type (Fig. 5K). These results suggest that *abo8-1* suffers from a stronger mitochondrial stress than the wild type.

### ABA inhibits root growth by regulating root meristem activity

**Figure 6:**
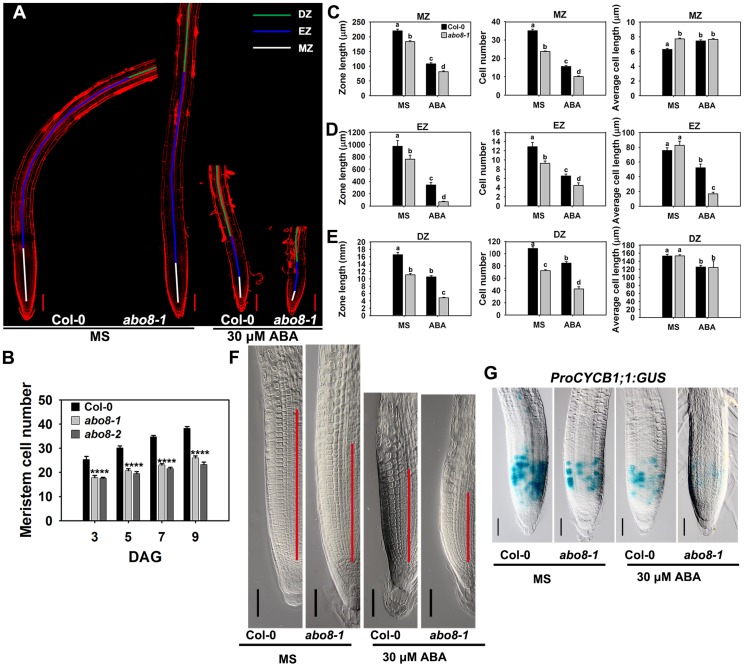
*abo8* mutation decreases the activity of the root meristem. A. The meristem zone (white line), elongation zone (blue line), and differentiation zone (green line) with 0 or 30 µM ABA treatment. Bars = 100 µm. Each image was made by joining several photographs of the same root. B. Meristem cell number of the wilt type, *abo8-1* and *abo8-2* in different times after seed germination (DAG). C. The meristem zone length, cell number, and cell length with 0 or 30 µM ABA treatment. D. The elongation zone length, cell number, and cell length with 0 or 30 µM ABA treatment. E. The differentiation zone length, cell number, and cell length with 0 or 30 µM ABA treatment. In C–E, two experiments were done with similar results, each with three repeats, each repeat with 10 roots. Values are means ±SE from one experiment. Means with different letters are significantly different at P<0.01. F. Size of the amplified root meristem in the wild type and *abo8-1* with and without ABA treatment. Bars = 50 µm. G. The expression of *ProCYCB1;1:GUS* in the wild type and *abo8-1* with or without 30 µM ABA treatment. Bars = 50 µm.

Root growth is determined by the coordination of stem cell activity and the differentiation of the progeny cells. In the roots, three developmental zones can be recognized according to cell types: the differentiation zone (DZ), the elongation zone (EZ), and the meristem zone (MZ) (Fig. 6A). In normal growth condition, *abo8-1* and *abo8-2* had less MZ cell numbers than the wild type after seed germination for different days (Fig. 6B). We further measured the zone length and also the cell number and cell length in each zone with ABA treatment and without ABA treatment. The MZ was shorter and contained fewer but slightly longer cells in *abo8-1* than in the wild type in the absence of ABA treatment (Fig. 6A, 6C, 6F). ABA treatment reduced MZ length and cell number in both *abo8-1* and the wild type (Fig. 6A, 6C, 6F). ABA treatment did not change MZ cell length in *abo8-1* but increased a little MZ cell length in the wild type, so that MZ cell length became comparable in the wild type and *abo8-1*. The EZ was shorter and had fewer cells in *abo8-1* than in the wild type with and without ABA treatment. Although the EZ cell length was similar in *abo8-1* and the wild type without ABA treatment, ABA treatment reduced EZ cell length more in *abo8-1* than in the wild type (Fig. 6A, 6D). The DZ was shorter and had fewer cells in *abo8-1* than in the wild type, but DZ cell length was similar in *abo8-1* and in the wild type with and without ABA treatment (Fig. 6A, 6E). These results indicate that the *abo8* mutation greatly impairs root meristem activity, reduces cell number in three zones, as well as reduces cell length in EZ. The number of cells that expressed *ProCYCB1;1:GUS*, a reporter for G2 in the mitotic phase of cell cycle, was consistently much lower in *abo8-1* than in the wild type, suggesting that ABO8 promotes mitosis (Fig. 6G).

### Addition of the reducing agent GSH largely recovers the ABA-hypersensitive phenotype of *abo8-1*

**Figure 7:**
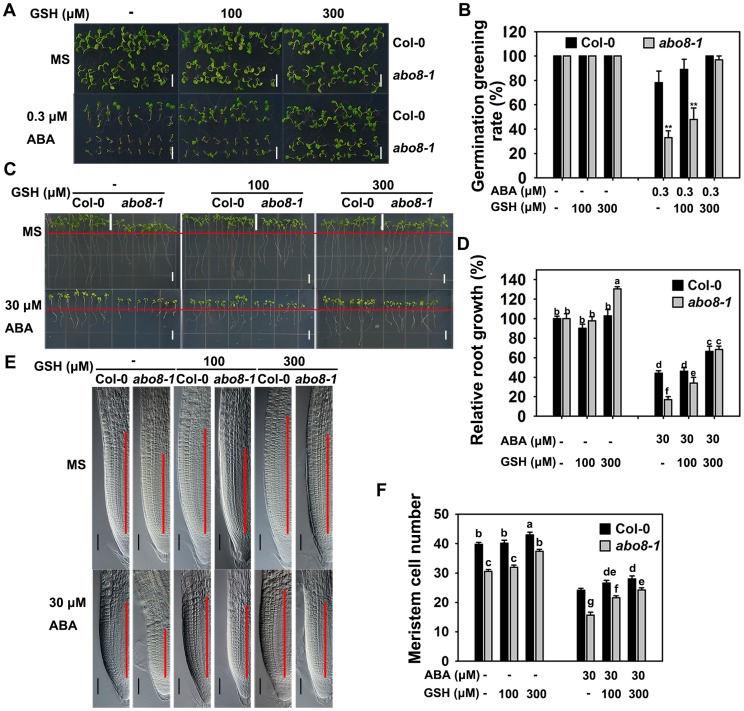
Addition of GSH partially recovers the ABA-hypersensitive phenotypes of *abo8-1* mutants. A. Germination greening of wild-type and *abo8-1* seeds on MS medium or MS medium containing 0.3 µM ABA, 0.3 µM ABA plus 100 µM GSH, or 0.3 µM ABA plus 300 µM GSH. Bars = 5 mm. B. Statistical analysis of the seed germination greening rate in (A). Three independent experiments were done with similar results, each with three replicates. About 20 seeds were counted from one plate as one replicate. Values are means ±SE from one experiment. **: P<0.01. C. Roots of wild-type and *abo8-1* seedlings grown on MS medium or MS medium containing 30 µM ABA, 30 µM ABA plus 100 µM GSH, or 30 µM ABA plus 300 µM GSH. The red line indicates the starting point of root growth after transfer. Bars = 5 mm. D. Statistical analysis of root growth in (C). Root growth is expressed relative to that of the wild type or *abo8-1* in MS medium without supplement. Three independent experiments were done with similar results, each with three replicates. Eight roots were measured from one plate as one replicate. Values are means ±SE from one experiment. Means with different letters are significantly different at P<0.01. E. Root meristem zones of seedlings grown on MS medium or MS medium containing 30 µM ABA, 30 µM ABA plus 100 µM GSH, or 30 µM ABA plus 300 µM GSH. Bars = 50 µm. F. Statistical analysis of the MZ cell number in (E). Three independent experiments were done with similar results, each with three repeats, and each repeat with 15–20 roots. Values are means ±SE from one experiment. Means with different letters are significantly different at P<0.01.

Because *abo8-1* accumulates more ROS than the wild type, we wanted to determine whether addition of a reducing agent would rescue the ABA-sensitive phenotype of *abo8-1*. Addition of different concentrations of the reducing agent GSH compromised the ABA-hypersensitive phenotype of *abo8-1* in both seed germination and root growth (Fig. 7A–7D). The seed germination greening ratio and relative root growth of *abo8-1* became comparable to those of the wild type following treatment with ABA plus 300 µM GSH (Fig. 7A–7D). Addition of 300 µM GSH increased the MZ cell number more in *abo8-1* than wild type with or without ABA treatment (Fig. 7E, 7F). These results indicate that reducing the oxidation status by addition of GSH decreased the ABA sensitivity of *abo8-1* in both seed germination and root growth.

### The expression of *ProDR5:GUS* in root tips is reduced in *abo8-1*, which can be partially reversed by GSH treatment

**Figure 8:**
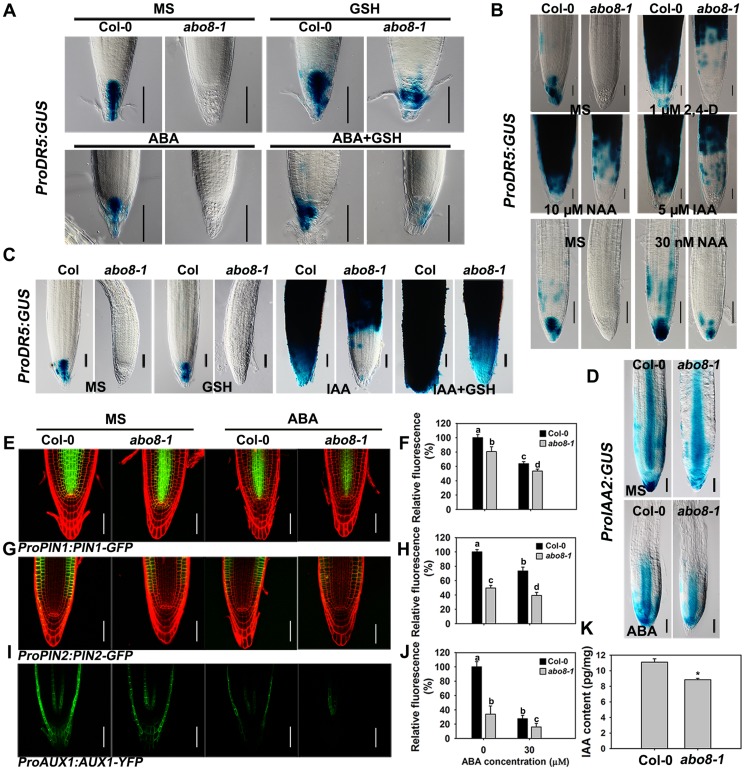
Expression of *DR5*, *IAA2*, *PIN1*, *PIN2*, and *AUX1* is altered in the *abo8-1* mutant. A. The expression of *ProDR5:GUS* in roots of seedlings grown on MS medium or MS medium containing 30 µM ABA, 300 µM GSH, or 30 µM ABA plus 300 µM GSH for 5 days. Bars = 50 µm. B. The expression of *ProDR5:GUS* in 5-day old seedlings treated in liquid MS medium without or with 1 µM 2,4-D, 5 µM IAA, and 10 µM NAA, or 30 nM NAA for 18 h. Bars = 50 µm. C. The expression of *ProDR5:GUS* in 5-day old seedlings treated in MS liquid medium without or with 300 µM GSH, 5 µM IAA, or 5 µM IAA plus 300 µM GSH for 18 h. Bars = 50 µm. D. The expression of *ProIAA2:GUS* with and without ABA treatment. Bars = 50 µm. E. The expression of *ProPIN1:PIN1-GFP* with and without ABA treatment. Bars = 50 µm. F. The fluorescence of PIN1-GFP in (E). Fluorescence is expressed relative to that of the wild type without ABA. Three experiments were done with similar results, each experiment with three repeats (each repeat with 15 roots). Values are means ±SE from one experiment. Means with different letters are significantly different at P<0.01 for a and b and at P<0.05 for c and d. G. The expression of *ProPIN2:PIN2-GFP* with and without ABA treatment. Bars = 50 µm. H. The fluorescence of PIN2-GFP in (G). Fluorescence is expressed relative to that of the wild type without ABA. Three experiments were done with similar results, each experiment with three repeats (each repeat with 15 roots). Values are means ±SE from one experiment. Means with different letters are significantly different at P<0.01 except for c and d at P<0.05. I. The expression of *ProAUX1:AUX1-YFP* with or without ABA treatment. Bars = 50 µm. J. The fluorescence of AUX1-YFP in (I). Fluorescence is expressed relative to that of the wild type without ABA. Three experiments were done with similar results, each experiment with three repeats (each repeat with 15 roots). Values are means ±SE from one experiment. Means with different letters are significantly different at P<0.01 except for b and c at P<0.05. K. The *abo8-1* contained less auxin than wild type. 7-day seedlings were used for auxin contents measurement. Each value is the mean ± SD (n = 4). *: P<0.05.

Because the activity of the root meristem is closely correlated with auxin signaling, the reduced root meristem activity in *abo8-1* is likely due to an impairment in auxin response. We introduced *ProDR5:GUS* and *ProIAA2:GUS* into *abo8-1* to check their expression levels. Both *ProDR5:GUS* and *ProIAA2:GUS* can be strongly induced by auxin [24], [25], [26]. The expression of *ProDR5:GUS* was strong in the root tips of the wild type in absence of ABA but was decreased by ABA treatment. However, GUS staining was barely detected in the root tips of *abo8-1* (Fig. 8A). When seedlings were moved from MS medium to MS medium supplemented with 300 µM GSH for 5 days, the *ProDR5:GUS* expression was detected in the root tips of *abo8-1*, which was largely abolished by addition of ABA. This result indicates that the low expression of *ProDR5:GUS* in *abo8-1* is likely due to the high oxidation status in root tips. GSH treatment also increased *ProDR5:GUS* expression in the wild type (Fig. 8A). When roots were treated with different auxins in high concentration, *ProDR5:GUS* expression was greatly induced in most parts of the wild-type root meristem, but only in the upper part of the *abo8-1* root meristem. However, when treated with low concentration of auxin (30 nM NAA), *ProDR5:GUS* expression could be detected in root tips of *abo8-1*, but lower than in the wild type. These results suggest that the *abo8-1* mutation reduces the auxin response around the root stem cell niche (Fig. 8B), and at low concentration of auxin, the oxidation status in root tips in *abo8-1* is compromised. In order to see whether reducing oxidation status by GSH could recover the *ProDR5:GUS* expression in auxin treatment, we treated the liquid growing seedling with GSH, IAA or GSH plus IAA. During a short time (18 h) treatment, GUS staining in the root tips of *abo8-1* was not able to be detected with IAA or GSH treatment alone, but could be clearly detected with IAA plus GSH treatment (Fig. 8C), suggesting that GSH changes the oxidative status in the root stem cell niche of *abo8-1*. However, the intensity of GUS staining was much lower in *abo8-1* than in the wild type. Similarly, the expression of *ProIAA2:GUS* was higher in the wild type than in *abo8-1* at both high and low concentration auxin treatments (S1 Figure), and although ABA treatment reduced its expression in the wild type and *abo8-1*, its expression was still higher in the wild type than in *abo8-1* (Fig. 8D).

Auxin transporters play crucial roles in modulating root growth. The expression of *ProPIN1:PIN1-GFP*, *ProPIN2:PIN2-GFP*, and *ProAUX1:AUX1-YFP* was much lower in *abo8-1* than in the wild type, and was reduced by ABA treatment in both *abo8-1* and the wild type (Fig. 8E and 8F for *ProPIN1:PIN1-GFP*, 8G and 8H for *ProPIN2:PIN2-GFP*, 8I and 8J for *ProAUX1:AUX1-YFP*). As both auxin level and signaling control the expression of auxin responsive genes, we measured the auxin contents in *abo8-1* and the wild type seedlings. The total auxin contents in *abo8-1* was ∼9.32 pg/mg, comparing with ∼10.83 pg/mg in wild type (P<0.05) (Fig. 8K), indicating that *abo8-1* mutation reduces the auxin content. These results indicate that both auxin level and response are largely impaired in *abo8-1*.

### The *abo8-1* mutation reduces the expression of *PLT1* and GSH treatment changes the distribution range of PLT1 protein in *abo8-1*

**Figure 9:**
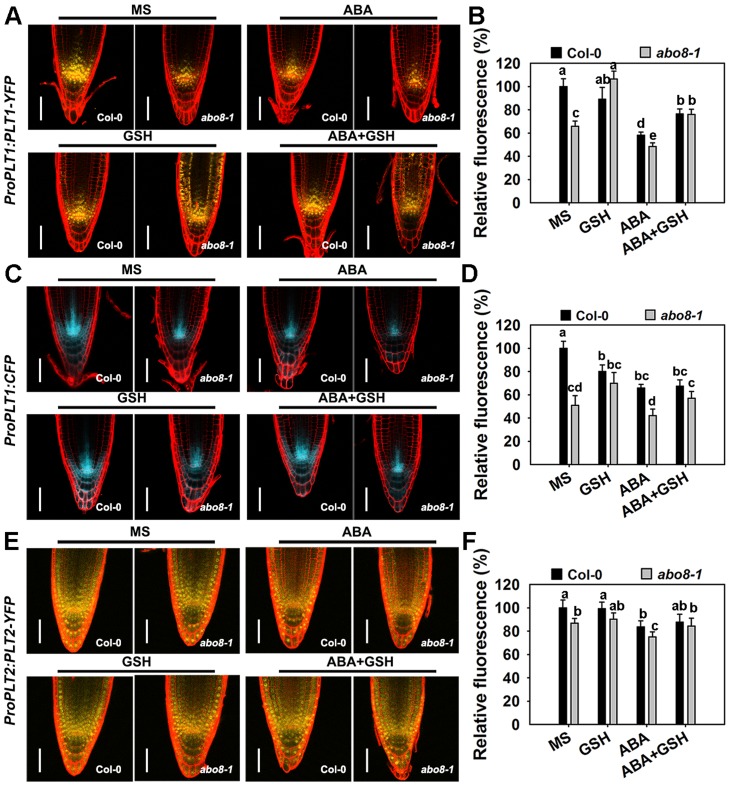
The *abo8* mutation affects the expression of *PLT1* and *PLT2*. A. The expression of *ProPLT1:PLT1-YFP* on MS medium or MS medium supplemented with ABA, GSH, or ABA plus GSH. Bars = 50 µm. B. The fluorescence of PLT1-YFP in (A). Fluorescence is expressed relative to that of the wild type in MS without supplement. Three independent experiments were done with similar results, each with three replicates. In each replicate, 8–15 roots were measured from one plate. Values are means ±SE from one experiment. Means with different letters are significantly different at P<0.01, except for c and d, d and e, at P<0.05. C. The expression of *ProPLT1:CFP* on MS medium or MS medium supplemented with ABA, GSH, or ABA plus GSH. Bars = 50 µm. D. The relative fluorescence of *ProPLT1:CFP* in (E). Fluorescence is expressed relative to that of the wild type in MS without supplement. Three independent experiments were done with similar results, each with three replicates. In each replicate, 8–15 roots were measured from one plate. Values are means ±SE from one experiment. Means with different letters are significantly different at P<0.01, except for a and b, c and d, at P<0.05. E. The expression of *ProPLT2:PLT2-YFP* on MS medium or MS medium supplemented with ABA, GSH, or ABA plus GSH. Bars = 50 µm. F. The relative fluorescence of PLT2-YFP in (C). Fluorescence is expressed relative to that of the wild type in MS without supplement. Three independent experiments were done with similar results, each with three replicates. In each replicate, 8–15 roots were measured from one plate. Values are means ±SE from one experiment. Means with different letters are significantly different at P<0.05.

The activity of the root meristem is regulated by different factors, among which the auxin-inducible *PLETHORA* (*PLTs*) are master regulators of root development [27], [28]. PLT1 and PLT2 are predominantly localized in the QC and redundantly regulate the root stem cell niche [28]. The transcriptional gradients of *PLT1* and *PLT2* strongly correlate with the auxin gradients in root apical meristem (RAM). *plt1 plt2* double mutants show loss of stem cells, loss of transit-amplifying cells, and reduced cell expansion in RAM [28]. As shown in Fig. 9A and 9B, the expression of *ProPLT1:PLT1-YFP* was much lower in *abo8-1* than in wild type both with and without ABA treatment. ABA treatment significantly reduced the expression of *ProPLT1:PLT1-YFP* in both *abo8-1* and the wild type. GSH treatment reduced a little strength of YFP fluorescence in the wild type, but apparently increased the expression of *ProPLT1:PLT1-YFP* in *abo8-1*. Interestingly, GSH treatment changed the distribution range of PLT1-YFP in *abo8-1*. Besides around root stem cell niche where PLT1-YFP is mainly localized, PLT1-YFP localization was shifted to upper parts in epidermis, cotex and endodermis. In the wild type, weak PLT1-YFP signals were also detected in epidermis above root stem cell niche by GSH treatment. In response to treatment with ABA plus GSH, the expression of *ProPLT1:PLT1-YFP* in *abo8-1* or the wild type was higher than only with ABA treatment (Fig. 9A, 9B). Again, the distribution of PLT1-YFP in epidermis, cotex and endodermis above root stem cell niche still existed in treatment with GSH plus ABA in *abo8-1*. In order to see whether PLT1 expression is affected by GSH at transcriptional level or not, we introduced *ProPLT1:CFP* into *abo8-1* by crossing the transgenic wild type plants that carry *ProPLT1:CFP* with *abo8-1*. The expression of *ProPLT1:CFP* was lower in *abo8-1* than the wild type (Fig. 9C, 9D). ABA treatment reduced the expression of *ProPLT1:CFP* in both *abo8-1* and the wild type, but the expression level of *ProPLT1:CFP* was still higher in the wild type than *abo8-1*. GSH treatment reduced a little the expression level of *ProPLT1:CFP* in wild type, but increased in *abo8-1*, which is consistent with expression of *ProPLT1:PLT1-YFP* under GSH treatment. However, *ProPLT1:CFP* expression domain was only found around root stem cell niche, not like the expression of *ProPLT1:PLT1-YFP* by GSH treatment in *abo8-1*. These results indicate that GSH treatment does not affect the expression area of *ProPLT1:CFP* at transcriptional level. It is likely that GSH treatment leads to more stability of PLT1-YFP in epidermis, cotex and endodermis above root stem cell niche in *abo8-1* than the wild type, suggesting that the *abo8-1* mutation disturbs the redox homeostasis which cannot be completely reversed by GSH treatment, and PLT1-YFP distribution in root tips is influenced by redox homeostasis.

The expression of *ProPLT2:PLT2-YFP* was also lower in *abo8-1* than in the wild type, and the expression of *ProPLT2:PLT2-YFP* was further reduced by ABA treatment in both genotypes (Fig. 9E, 9F). However, addition of GSH did not clearly affect the expression of *ProPLT2:PLT2-YFP* in *abo8-1* and the wild type. With ABA treatment, GSH slightly increased expression of *ProPLT2:PLT2-YFP* in *abo8-1* and the wild type. These results suggest that PLT2 is less affected by the *abo8-1* mutation and ABA treatment than PLT1.

### *plt1* and *plt2* mutants are sensitive to ABA in root growth

**Figure 10:**
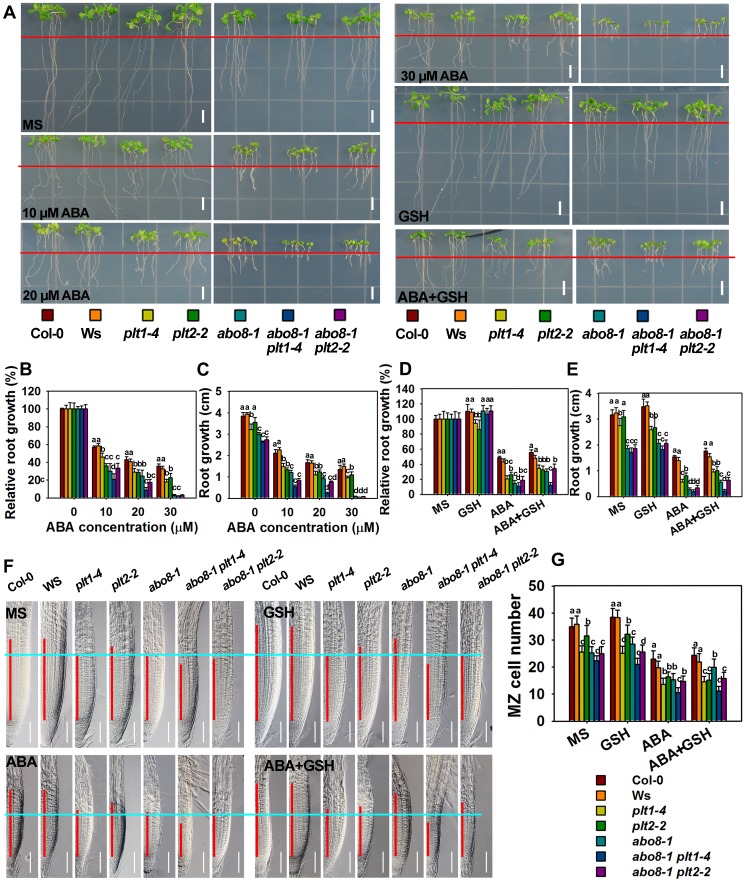
Genetic analysis of *ABO8* with *PLT1* and *PLT2*. A. The roots of the wild type Col-0, Ws, *plt1-4*, *plt2-2*, *abo8-1*, *abo8-1 plt1-4*, and *abo8-1 plt2-2* grown on MS medium or MS medium supplemented with different concentrations of ABA, 300 µM GSH or 30 µM ABA plus 300 µM GSH for 5 days. Red line indicates the root tip positions after 5-day seedlings were just transferred onto different medium. Bars = 5 mm. B–E. The root growth and relative root growth of different plants in (A). Relative root growth is expressed to that of the wild type or mutants on MS without ABA. About 15 roots from three plates were measured in each experiment, and three experiments were done with similar results. Values are means ±SE, n = 3. Means with different letters are significantly different at P<0.01. F. The length of the root meristem (red line) of different genotypes on MS medium or MS medium supplemented with 30 µM ABA, 300 µM GSH or 30 µM ABA plus 300 µM GSH. Bars = 50 µm. G. The MZ cell number in (F). Three independent experiments were done with similar results, and each experiment had three replicates. About 20 roots were measured for each replicate. Values are means ±SE from one experiment. Means with different letters are significantly different at P<0.01.

To determine whether PLT1 and PLT2 are involved in ABA inhibition of root growth, we examined the ABA sensitivity of *plt1* and *plt2* mutants. The root growth of *plt1-4* and *plt2-2* [28] was more sensitive to ABA than the wild type (Fig. 10A–E), suggesting that PLT1/2 are negative regulators in ABA-mediated root growth. The root length of the *abo8-1 plt1-4* or *abo8-1 plt2-2* double mutant was a little shorter than that of *abo8-1*, *plt1-4* or *plt2-2* in the absence of ABA treatment, suggesting that PLT1 or PLT2 and ABO8 have additive function in root growth. ABA treatment at 10 or 20 µM inhibited root growth more in *abo8-1 plt1-4* and *abo8-1 plt2-2* than in *abo8-1*, *plt1-4* or *plt2-2*, suggesting that PLT1/2 and ABO8 have additional function in ABA inhibition of root growth. At 20 µM ABA, the root growth of *abo8-1plt1-4* was more inhibited than that of *abo8-1plt2-2*. At 30 µM ABA, the root growth of *abo8-1*, *abo8-1 plt1-4* and *abo8-1 plt2-2* was almost completely inhibited, and the root growth of *plt1-4* was more inhibited than that of *plt2-2*. These results indicate that PLT1 plays a more important role than PLT2 in ABA inhibition of root growth. We also found that addition of 300 µM GSH could partially recover the ABA inhibition of root growth for *plt1-4*, *plt2-2*, *abo8-1 plt1-4* and *abo8-1 plt2-2* mutants (Fig. 10A–E).

We further compared the cell number in the MZ among different mutants (Fig. 10F, 10G). *abo8-1* had similar MZ cell number as *plt1-4*, both of which were reduced to a similar level by ABA treatment. *abo8-1 plt1-4* double mutant had less MZ cell number than *abo8-1* or *plt1-4* without or with ABA treatment, suggesting that PLT1 and ABO8 have additive function in regulating MZ. *plt2-2* had less MZ cell number than the wild type, but more than *plt1-4* or *abo8-1* without ABA treatment, and only a little more MZ number than *abo8-1* or *plt1-4* with ABA treatment. *abo8-1 plt2-2* had similar MZ cell number as *abo8-1*, and much less than *plt2-2*. ABA treatment reduced the MZ cell number of *abo8-1 plt2-2* to a similar level as that of *abo8-1*. Interestingly, GSH treatment did not significantly increase the MZ cell number in *plt1-4*, *plt2-2*, *abo8-1plt1-4*, and *abo8-1 plt2-2* with ABA or without ABA treatment. However, GSH treatment clearly increased MZ cell number in the wild type and *abo8-1*. These results suggest that both PLT1 and PLT2 play crucial roles in ABA inhibition of root growth, and PLT1 is more important than PLT2 in this process.

**Figure 11:**
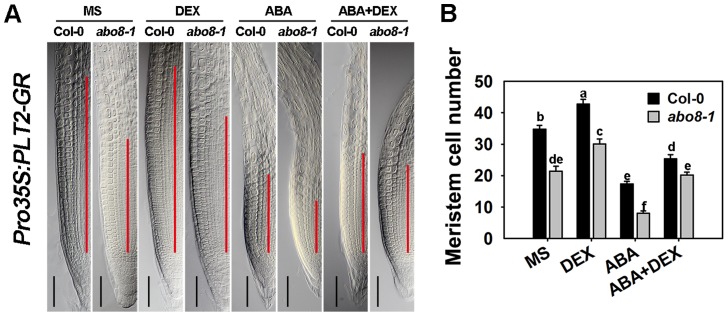
The sensitivity of *abo8-1* roots to ABA is partially rescued by the expression of the inducible *Pro35S:PLT2-GR*. A. The length of the root meristem (red line) of wild-type (carrying *Pro35S:PLT2-GR*) and *abo8-1* (carrying *Pro35S:PLT2-GR*) seedlings on MS medium or MS medium supplemented with DEX, ABA, or ABA plus DEX. Bars = 50 µm. B. Root meristem cell number of the wild type (Col-0) and *abo8-1* in (A). Three independent experiments were done with similar results, and each experiment had three replicates. About 20 roots were measured for each replicate. Values are means ±SE from one experiment. Means with different letters are significantly different at P<0.01.

To determine whether PLT1/2 can partially complement the ABA hypersensitivity of *abo8-1* in root growth, we introduced an inducible *Pro35S:PLT2-GR* into *abo8-1* by crossing a *Pro35S:PLT2-Glucocorticoid Receptor* (*Pro35S:PLT2-GR*) transgenic plant with *abo8-1*. After dexamethasone (DEX) induction, the meristem cell number of the wild type was increased 23.1% comparing with 40.4% in *abo8-1* in the absence of ABA treatment (Fig. 11A, 11B). The reduced root meristem by ABA treatment was increased 151.5% in *abo8-1* comparing with 40.5% in wild type by DEX treatment. These results suggest that the retarded root growth of *abo8-1* is partially caused by low expression of *PLT* genes, which is likely due to reduced auxin accumulation and/or auxin signaling by the *abo8-1* mutation.

### The *abo8* mutation deregulates the differentiation of root distal stem cells (DSC)

**Figure 12:**
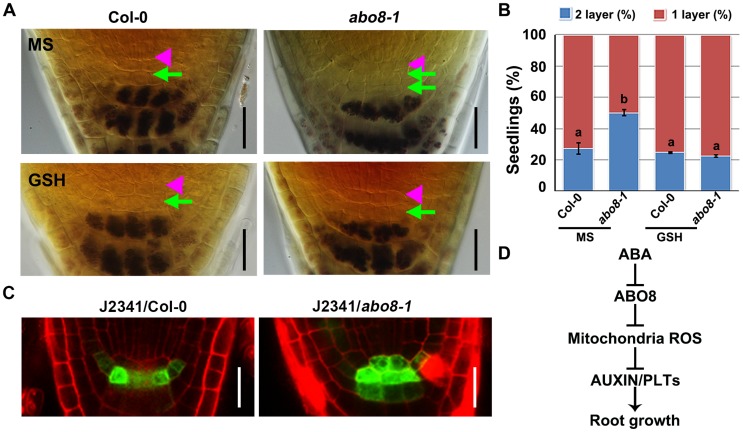
*abo8* mutation delays the distal stem cell (DSC) differentiation. A. Differentiation status of DSC in 5-day-old seedlings on MS medium or MS medium with 300 µM GSH. DSC (green arrowheads) are characterized as cells between the QC (pink triangle) and the starch containing differentiated columella cells. Bars = 20 µm. B. Quantitative evaluation of DSC layers in A. About 30 roots were examined for each biological repeat, three experiments were done with similar results. Values are means ±SE from one experiment. Means in different letters are P<0.01. C. The expression of DSC marker J2341 in roots of the wild type and *abo8-1* mutants. Bars = 20 µm. D. A proposed model for ABO8 in root growth. ABO8 is downreguated by ABA. ABO8 negatively modulates mitochondria ROS, and ROS negatively modulate auxin level and signaling to control PLTs expression. It is likely that ROS also directly regulate PLT1 as posttranscriptional level.

Root growth is determined by quiescent center (QC) and its surrounding stem cells [29]. The distal stem cells (DSC) below the QC generates the columella cells with distinct starch granules [30]. In the root stem cell niche, about 25% wild type roots have two layers of DSC comparing with 50% *abo8-1* roots as they do not contain starch and cannot be stained by Lugol's solution (Fig. 12A, 12B). Addition of GSH did not change the ratio of two DSC layers in the wild type, but reduced two DSC layers from 50% to 25% in *abo8-1* (Fig. 12A, 12B). These results suggest that GSH antagonistically mediates the inhibition of root DSC differentiation by the *abo8* mutation.

We also introduced J2341 marker, a specific DSC marker [31], into *abo8-1* to observe GFP localization. GFP fluorescence was observed in one layer of DSC in near 75% wild-type roots comparing with only about 25% in *abo8-1* roots (20 roots were accounted each time, repeated two times) (Fig. 12C). These results indicate that the *abo8-1* mutation delays the DSC differentiation.

## Discussion

Recent studies have indicated that a plant-specific family of PPR proteins is involved in crucial RNA metabolism in plastids and mitochondria [32]. In this study, we found that ABO8, a PPR protein localized in mitochondria, is specifically required for the splicing of *NAD4* intron 3 in mitochondrial complex I. A defect of ABO8 leads to various retarded growth phenotypes, high accumulation of ROS, and ABA hypersensitivity in seed germination and root growth. We further showed that the ABA hypersensitivity in root growth of *abo8* mutants is due to a high accumulation of ROS that decreases auxin accumulation and signaling, which results in greatly reduced expression of *PLT* genes (Fig. 12D). Overexpressing *PLT2* in an inducible system can largely recover the root-hypersensitive phenotype of *abo8* under ABA treatment, suggesting that PLTs are important in the ABA inhibition of root growth.

Plant cells contain two kinds of symbiotic organelles, mitochondria and plastids, both of which have their own genomes that encode a limited number of proteins [32]. Although the mitochondrial genome contains only about 40 genes, >2000 proteins have been identified in mitochondria [33]. The mitochondrial protein-coding genes must be finely regulated by many different nuclear proteins, most of which are PPR proteins. The following PPR proteins have been found to facilitate *NAD4* mRNA metabolism in *Arabidopsis*: MTSF1 (mitochondrial stability factor 1) for the 3′-processing of *NAD4* mRNA and its stability [34]; RPF1 (RNA PROCESSING FACTOR 1) for 5′-end processing of the *NAD4* mRNA [35]; AHG11 (ABA hypersensitive germination 11) for *NAD4* RNA editing [36]; SLO1 (SLOW GROWTH1) for editing *NAD4* and *NAD9* [37]; LOI1 (LOVASTATIN INSENSITIVE 1) for editing *NAD4*, *CCB203*, and *COX3* [38], [39]; and ABO8 for splicing of *NAD4* intron 3. Except for PPR proteins, a mutation in the group II intron-encoded reverse transcriptase/maturase (At-nMat1a) also impairs *cis*-splicing of *NAD4* intron 2 and *NAD2* intron 1 and *trans*-splicing of *NAD1* intron1 [40], [41], [42].

A dysfunction in the mitochondrial electron transport chain of complex I can cause a redox imbalance and increases in ROS accumulation. Mutants with impaired mitochondrial functions usually have various severe phenotypes including retarded seedling growth and abiotic stress responses. Several mutants such as *abo5* [21], *abo6* [19], *ahg11* [36], *slo2* (for editing several transcripts in complex I) [43], and *slg1* (editing *NAD3* transcript) [44] accumulate more ROS than the wild type and are more sensitive to ABA than the wild type in seed germination and/or root growth. In a previous study, we found that ROS accumulation in *abo6* impairs auxin accumulation and/or signaling, suggesting that a redox regulatory mechanism plays crucial roles in controlling root growth [19]. The *abo8* mutants have the similar retarded growth phenotype and ABA sensitivity as *abo6* or *abo5* and also accumulate more ROS than the wild type. Addition of the reducing agent GSH can restore the ABA sensitivity in both root growth and seed germination, probably by reducing ROS. Root tips and lateral root primordia are the regions where auxin is highly accumulated. Recent studies indicate that the bioactive IAA can be irreversibly oxidized into OxIAA (2-oxindole-3-acetic acid), a form that has little auxin activity [20], [45]; this process mainly occurs in the root tips [20]. Oxidation of auxin to OxIAA is an important mechanism for removing high levels of active auxin from the root apex to maintain auxin homeostasis [20]. Our study indicates that ROS are highly accumulated in root tips and that such accumulation might facilitate the oxidation of IAA in these tissues. Although a highly oxidized status is important for maintaining the low mitotic activity in QC cells [46], greatly increased ROS accumulation caused by mutations such as *abo8* and *abo6* would disrupt the redox homeostasis, further oxidize IAA, reduce the IAA level, and ultimately reduce the stem cell activity. The root meristems are much shorter in *abo8-1* than in the wild type. Interestingly, in this study, we found that addition of high concentrations of auxins (IAA or NAA) did not induce the expression of auxin responsive marker gene *ProDR5:GUS* around root stem cell niche, but only highly induced its expression in upper part of root meristem in *abo8-1*, while in wild type *ProDR5:GUS* was highly induced in the whole root meristem. However, addition of auxins plus GSH was able to induce the expression of *ProDR5:GUS* in the whole root meristem. These results suggest that redox homeostasis plays crucial roles in maintaining a proper auxin gradient and auxin response in root tips.

*PLT* genes are essential for regulating the patterning of root stem cell niche [27], [28]. The expression domains of *PLT* overlap with the auxin gradient in the root tips. The auxin gradient is thought to control root growth by tightly regulating the expression of *PLTs* [27], [28]. In this study, we found that the *plt1-4* and *plt2-2* mutants are more sensitive to ABA than the wild type in root growth, suggesting that PLTs are negative regulators in ABA inhibition of root growth. In respect to regulating RAM, the *abo8-1* mutation enhances the phenotypes of *plt1-4* and *plt2-2*, suggesting that PLT1/PLT2 and ABO8 additionally regulate root growth. Interestingly, GSH treatment does not change the expression pattern of *ProPLT1:CFP* around root stem cell niche, but does change the distribution of PLT1-YFP in *abo8-1*. PLT1-YFP is usually localized around root stem cell niche in the wild type, but in *abo8-1*, besides around the root stem cell niche is also found above it. These results suggest that distribution of PLT1 protein might be affected by both redox homeostasis and auxin response in root tips. The previous study on *miao* mutant indicates that the *miao* weak mutation in GR2 (GSH reductase) greatly reduces GSH content, and retards root growth [15]. The expression of *PLT1* and *PLT2* is decreased in the *miao* mutant. Interestingly, introducing a dexamethasone (DEX)-inducible *PLT2* into the *miao* mutant with overproduction of PLT2 does not increase the RAM length of the *miao* mutant, suggesting that a reduced glutathione environment is required for proper function of PLT downstream factors in root meristem. However, the DEX-inducible PLT2 can largely induce the RAM expansion of *abo8-1*, suggesting that PLT2 acts downstream of ABO8 for RAM maintenance. The different RAM responses of *miao* and *abo8-1* to PLT2 overexpression suggest that oxidation status imposed by high accumulation of ROS from impairment of mitochondria and glutathione redox status by reduced GSH level have different effects on PLTs/Auxin signaling. *abo8* mutation also reduces the expression of *PINs* and *AUX1*, and delays DSC differentiation. Taken together, these results demonstrate that ROS from mitochondria are crucial mediators for root growth.

## Materials and Methods

### Plant materials and growth conditions

Surface-sterilized wild type *Arabidopsis thaliana* (Col-0) seeds were plated on MS medium containing 1% sucrose and 0.8% agar. The plates were stratified at 4°C for 2 days and then transferred to a light incubator (22°C) with 22 h light/2 h dark. For the seed germination greening assay, the seedlings were grown for 7 days before photographed. For the root growth assay, 4-day-old seedlings were transferred to MS or MS medium supplemented with different concentrations of ABA or GSH. The root tips were placed in a straight line. The plates were oriented vertically in a light incubator for another 5 days before they were photographed. Root growth was measured with Imag J (Image J; National Institutes of Health; http://rsb.info.nih.gov/ij). In a greenhouse, the seedlings were grown at 22°C and 16 h light/8 h dark under 50 µmol m^−2^ s^−1^ light. ABA (Sigma-Aldrich) was prepared as a 30 mM stock solution in ethanol. *plt1-4* and *plt2-2* in *Arabidopsis thaliana* ecotypes Wassilewskija (Ws) were used for genetic analysis [28].

### Mutant screening

*Arabidopsis* (Col-0) seeds were mutated with EMS (ethyl methanesulfonate). The 5-day-old M2 seedlings were transferred to MS medium containing 30 µM ABA with the root tips upward and were grown for another 7 days. The seedlings with short roots were selected as putative mutants. The ABA-sensitive phenotype of the putative mutants was rechecked by comparing the root growth on MS medium and MS medium supplemented with 30 µM ABA in the second generation.

### Map-based cloning and mutant complementation

*abo8-1* (Col-0) was crossed with Landsberg *erecta*. A total of 912 *abo8-1* seedlings were selected from the segregating F2 population for map-based cloning. *ABO8* was localized between two SNP makers on bacterial artificial chromosomes F25E4 and T26M18 in chromosome 4. This region contains genes numbered from *AT4G11390* to *AT4G11876* (The TAIR SeqViewer; http://www.arabidopsis.org/servlets/sv). We sequenced all of the open reading frames in this region and only identified a G447 to A447 (counting from the first putative ATG) mutation, which caused a premature stop codon in *AT4G11690*.

The genomic sequence containing a complete and single gene, *AT4G11690*, was amplified with the following primers: 5′-CGCGGATCCCGGCATTTGATCTATGTC GTTCTTC-3′ and 5′-GAGGGTACCTTTTGAGCTTACATGTGAATCGTTTTTGG-3′. The DNA fragment was cloned in the *Bam*HI and *Kpn*I sites of a modified vector pCAMBIA1300, with the C-terminus of ABO8 fused with green fluorescent protein (GFP). The construct *ProABO8:ABO8-GFP* was then introduced into *abo8-1* via *Agrobacterium*-mediated transformation. The transgenic plants were screened on MS medium containing 30 mg/L hygromycin.

We obtained a T-DNA line SALK_025470 with T-DNA inserted at +358 bp of the *AT4G11690* gene from the Arabidopsis Biological Resource Center (ABRC; http://abrc.osu.edu).The homozygous T-DNA insertion line was obtained using the following primers: LP, 5′-CTGCATAAACTCAACGCCTTC-3′; RP, 5′-TTTAAACGTATCTGCCATGGC-3′; TF, 5′-ATTTTGCCGATTTCGGAAC-3′. *abo8-1* was crossed with homozygous SALK_025470, and the F1 plants were analyzed for root sensitivity to ABA.

### Subcellular localization

The roots of 10-day-old seedlings of homozygous transgenic line *ProABO8:ABO8-GFP* were imaged using a confocal laser scanning microscope (LSM510; Carl Zeiss, Oberkochen, Germany). About eight independent transgenic lines expressing the *ProABO8:ABO8-GFP* were analyzed. The iPSORT (http://ipsort.hgc.jp) program predicts that the PPR protein ABO8 is targeted to the mitochondrion. To study the subcellular localization of ABO8, we prepared protoplasts from the roots of 10-day-old *ProABO8:ABO8-GFP* seedlings, and mitochondria were stained with MitoTracker orange as a control for co-localization. The images were obtained using a Carl Zeiss LSM510 META laser scanning microscope. GFP signals were collected using emission filter BP505–530 nm with an excitation at 488 nm, and red signals (MitoTracker stain) were obtained using BP 585–615 nm with an excitation at 543 nm.

### GUS staining

The *ABO8* promoter (about 2.1 kb) was cloned into pCAMBIA1391 (with a GUS coding region) between the *Bam*HI and *Spe*I sites using the following primers: 5′-CGCGGATCCCGGCATTTGATCTATGTCGTTCTTC-3′ and 5′-CGCGACTAGTGTGTGTGTTGAAATTCATGGATTCG-3′. The *ProABO8:GUS* construct was then transferred into Col-0 via *Agrobacterium*-mediated transformation. About 20 independent transgenic lines expressing *ProABO8:GUS* were analyzed. For the GUS staining assay, seedlings were incubated in 0.1 M phosphate buffer (pH 7.0), 5 mM K_4_Fe(CN)_6_, 5 mM K_3_Fe(CN)_6_, 0.1% Triton X-100, and 0.5 mg/mL X-Gluc at 37°C in the dark for 24 h and were then incubated in 75% ethanol overnight. Seedlings were photographed with an Olympus BX53 microscope and an Olympus SZX16 stereoscopic microscope.

The transgenic plants carrying *ProCYCB1;1:GUS*, *ProDR5:GUS*,or *ProIAA2:GUS* were crossed with *abo8-1* mutants. The F2 seedlings were genotyped for the *abo8-1* allele using specific dCAPS primers: 5′-CCACTCCCAATTCTTCACATCTTCCTC-3′ and 5′-CTTTGCTTTTGTTCTCGTTGAAGAAGCT-3′. The PCR products were digested with *Hin*dIII. The homozygous transgenic lines of F3 plants were selected on MS containing 50 mg/L kanamycin. GUS staining was carried out as described earlier [19]. Staining time was 90 min for *ProCYCB1;1:GUS*, 50 min for *ProIAA2:GUS*, and 18 h for *ProDR5:GUS*.

### Northern blot and qRT-PCR

RNA was extracted from 10-day-old seedlings that were treated or not treated with 50 µM ABA for 5 h in liquid MS. A 20-µg quantity of RNA per sample was used for Northern blot as previously described [21].The probes used for Northern blot were described previously [21]. *TUBULIN4* was used as a loading control.

For qRT-PCR, 4 µg of total RNA was first digested with DNase I (TaKaRa) and then reverse transcribed into Poly(dT) cDNA using a Moloney Murine Leukemia Virus Transcriptase kit (Promega) according to the manufacturer's instructions. qRT-PCR was performed as previously described [19]. *ACTIN2* was used as the internal control. The primers designed to analyze *NAD4* splicing were described previously [19]. The primers used to analyze *ABO8* expression were gene specific primers that flanking the T-DNA insertion: 5′-GGTTTTGTTCCTGGATCGAATTGCTTCAAC-3′ and 5′-TCCCAAAGCTATACACGT CCAAAACAAC-3′. The primers designed to analyze *AOX1a* expression were described previously [44].

### Blue Native-PAGE and complex I activity assay

5-day-old seedlings were treated with or without 50 µM ABA for 24 h. The crude mitochondrial membrane proteins were prepared according to the method described previously [44]. 20 µg protein of each sample was loaded onto and separated by a 4.5% to 16% gradient Blue-Native PAGE (Invitrogen, BN1002BOX) according to the manufacturer's instructions. The activity of mitochondria complex I were analyzed by staining the gel in reaction buffer (50 mM Mops-KOH at pH 7.6, 0.2 mM NADH and 1 mM NBT). The reaction was stopped when the dark blue stain was strong enough by immersing the gel in 30% methanol and 10% acetic acid (v/v).

### Measurement of cellular ATP content

5-day-old seedlings were treated with or without 50 µM ABA for 24 h. About 100 mg seedlings was ground to powder in liquid nitrogen and resuspended in 400 µL of 2.3% (v/v) trichloroacetic acid and mixed vigorously. After centrifuged for 15 min at 20,000 g, the supernatant was collected and adjusted to pH 7.0 with 2.5 M K_2_CO_3_. Adenosine 5′-triphosphate (ATP) bioluminescent assay Kit (SigmaAldrich, fl-aa) was used to measure the ATP concentration according to the method described previously [47].

### Auxin content measurement

7-day-old seedlings of the wild type and *abo8-1* were harvested and ground to powder in liquid nitrogen. For each sample, about 50–100 mg powder were resuspended in pre-cooling 80% methanol and mix immediately. The samples were kept at 4°C protected from light before 0.8 ng [^13^C]-IAA was added. Free IAA content measurement was performed using GC-QQQ (Agilent, 7000A) in Institute of Botany, the Chinese Academy of Sciences.

### Measurement of ROS in plants

Four-day-old seedlings of Col-0 and *abo8-1* were treated in liquid MS with or without 50 µM ABA for 5 h before the seedlings were stained as described in the following paragraphs.

For 3′, 3′- diaminobenzidine (DAB) staining to detect H_2_O_2_, the seedlings were incubated in 0.3 mg/mL DAB (Sigma-Aldrich) dissolved in 50 mM Tris-HCl (pH 5.0) for 8 h. For nitroblue tetrazolium (NBT) staining to detect superoxides, the seedlings were incubated in a reaction buffer containing 1 mM NBT (Sigma-Aldrich), 20 mM K-phosphate, and 0.1 M NaCl at pH 6.2 for 15 min. The seedlings stained by DAB or NBT were then washed three times with water. For clearing, seedlings were incubated in acidified methanol buffer (10 mL of methanol, 2 mL of HCl, 38 mL of water) at 57°C for 15 min and then in a basic solution (7% NaOH in 60% ethanol) for 15 min at room temperature. The seedlings were incubated 10 min at each step in the following series: 40% ethanol, 20% ethanol, 10% ethanol, 5% ethanol, and 25% glycerol. The seedlings were then examined in 50% glycerol with an Olympus BX53 microscope.

For 2′,7′-dichlorodihydrofluorescin diacetate (DCFH-DA) staining to detect H_2_O_2_, the seedlings were incubated in a buffer containing 50 µM DCFH-DA (Sigma-Aldrich) and 20 µM K-phosphate at pH 6.0 in darkness for 10 min. The roots were then photographed using a Carl Zeiss LSM510 META confocal microscope with an excitation at 488 nm. The intensities of the fluorescent signals were statistically compared with the Student's *t*-test.

### Measurement of root meristems

For analysis of root meristems, 4-day-old seedlings were transferred to MS medium or MS medium supplemented with 30 µM ABA, 300 µM GSH, or 2 µM DEX. After 3 days, the roots were placed in a mounting solution (7.5 g gum arabic, 100 g chloroacetaldehyde, 5 mL glycerol, and 60 mL water) and were photographed with an Olympus BX53 microscope. We measured the meristem cell number in the cortex between the QC and the first elongating cell, elongation zone (EZ) cell number from the first elongating cell to first cell with a root hair. Differential zone is from the first cell with a root hair to the joint of root and hypocotyl.

### Starch staining

Seedlings were immersed into Lugol (0.2% iodine and 2% potassium iodine) and incubated for about 1 min, washed in water once and mounted on the slide with a mounting solution described above. The Lugol-stained seedlings were photographed with an Olympus BX53 microscope.

### Fluorescence microscopy

*Arabidopsis* transgenic plants harboring *ProPLT1:PLT1-YFP*, *ProPLT2:PLT2-YFP*, *ProPLT1:CFP*, *ProPIN1:PIN1-GFP*, *ProPIN2:PIN2-GFP*, *ProAUX1:AUX1-YFP*, J2341, or Mito-cpYFP [19] were crossed with *abo8-1*. The method for genotyping the *abo8-1* allele was described earlier. An Olympus SZX16 stereo fluorescence microscope was used to select the homozygous transgenic plants from the F3 generation. Four-day-old homozygous seedlings were transferred to MS medium or MS containing 30 µM ABA, 300 µM GSH, or both, and were grown for another 2 days. Propidium iodide fluorescence was used to visualize the cells in the root tip. Seedlings were incubated in 10 µM propidium iodide (Sigma-Aldrich) for 3 min, and the roots were then imaged with a Carl Zeiss LSM510 META confocal microscope. Fluorescence intensities were measured using AxioVision Rel. 4.8 software.

## Supporting Information

S1 Figure The expression of *ProIAA:GUS* in 5-day old seedlings treated in liquid MS medium without or with 1 µM 2,4-D, 5 µM IAA, and 10 µM NAA, or 30 nM NAA for 18 h. Bars = 50 µm.
(TIF)
