# Supplementary figures and images for: ABA-Mediated ROS in Mitochondria Regulate Root Meristem Activity by Controlling PLETHORA Expression in Arabidopsis
Source: PLoS Genet. 2014 Dec 18;10(12):e1004791. doi: 10.1371/journal.pgen.1004791 (PMC4270459; doi:10.1371/journal.pgen.1004791)

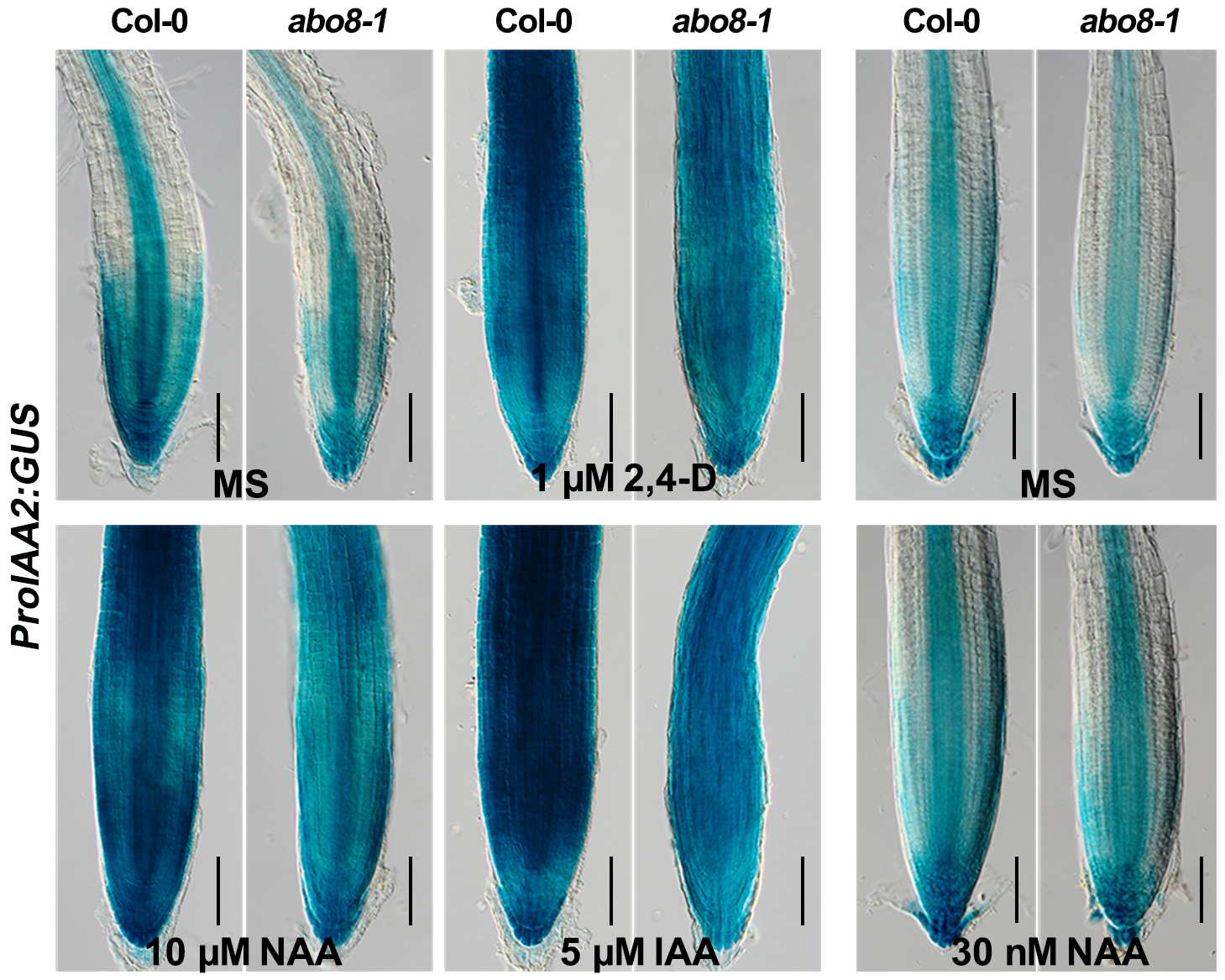

Supplement: S1 Figure — The expression of ProIAA:GUS in 5-day old seedlings treated in liquid MS medium without or with 1 µM 2,4-D, 5 µM IAA, and 10 µM NAA, or 30 nM NAA for 18 h. Bars = 50 µm. (TIF) [file pgen.1004791.s001.tif]
